# Characterization of *GUCA1A*-associated dominant cone/cone-rod dystrophy: low prevalence among Japanese patients with inherited retinal dystrophies

**DOI:** 10.1038/s41598-019-52660-1

**Published:** 2019-11-14

**Authors:** Kei Mizobuchi, Takaaki Hayashi, Satoshi Katagiri, Kazutoshi Yoshitake, Kaoru Fujinami, Lizhu Yang, Kazuki Kuniyoshi, Kei Shinoda, Shigeki Machida, Mineo Kondo, Shinji Ueno, Hiroko Terasaki, Tomokazu Matsuura, Kazushige Tsunoda, Takeshi Iwata, Tadashi Nakano

**Affiliations:** 1 Department of Ophthalmology, The Jikei University School of Medicine, Tokyo, Japan; 2 Department of Ophthalmology, Katsushika Medical Center, The Jikei University School of Medicine, Tokyo, Japan; 3 Division of Molecular and Cellular Biology, National Institute of Sensory Organs, National Tokyo Medical Center, Tokyo, Japan; 4 Division of Vision Research, National Institute of Sensory Organs, National Hospital Organization Tokyo Medical Center, Tokyo, Japan; 5 Department of Ophthalmology, Keio University School of Medicine, Tokyo, Japan; 6 UCL Institute of Ophthalmology associated with Moorfields Eye Hospital, London, UK; 7 Department of Ophthalmology, Kindai University Faculty of Medicine, Osaka, Japan; 8 Department of Ophthalmology, Teikyo University School of Medicine, Tokyo, Japan; 9 Department of Ophthalmology, Dokkyo Medical University Saitama Medical Center, Saitama, Japan; 10 Department of Ophthalmology, Iwate Medical University School of Medicine, Iwate, Japan; 11 Department of Ophthalmology, Mie University Graduate School of Medicine, Mie, Japan; 12 Department of Ophthalmology, Nagoya University Graduate School of Medicine, Aichi, Japan; 13 Department of Laboratory Medicine, The Jikei University School of Medicine, Tokyo, Japan

**Keywords:** Gene amplification, Hereditary eye disease

## Abstract

*GUCA1A* gene variants are associated with autosomal dominant (AD) cone dystrophy (COD) and cone-rod dystrophy (CORD). *GUCA1A*-associated AD-COD/CORD has never been reported in the Japanese population. The purpose of this study was to investigate clinical and genetic features of *GUCA1A*-associated AD-COD/CORD from a large Japanese cohort. We identified 8 variants [c.C50_80del (p.E17VfsX22), c.T124A (p.F42I), c.C204G (p.D68E), c.C238A (p.L80I), c.T295A (p.Y99N), c.A296C (p.Y99S), c.C451T (p.L151F), and c.A551G (p.Q184R)] in 14 families from our whole exome sequencing database composed of 1385 patients with inherited retinal diseases (IRDs) from 1192 families. Three variants (p.Y99N, p.Y99S, and p.L151F), which are located on/around EF-hand domains 3 and 4, were confirmed as “pathogenic”, whereas the other five variants, which did not co-segregate with IRDs, were considered “non-pathogenic”. Ophthalmic findings of 9 patients from 3 families with the pathogenic variants showed central visual impairment from early to middle-age onset and progressive macular atrophy. Electroretinography revealed severely decreased or non-recordable cone responses, whereas rod responses were highly variable, ranging from nearly normal to non-recordable. Our results indicate that the three pathogenic variants, two of which were novel, underlie AD-COD/CORD with progressive retinal atrophy, and the prevalence (0.25%, 3/1192 families) of *GUCA1A*-associated IRDs may be low among Japanese patients.

## Introduction

The guanylate cyclase activator 1A (*GUCA1A*) gene (OMIM *600364) encodes for guanylyl cyclase-activating protein 1 (GCAP-1), which is expressed in both rod and cone outer segments^1^ and regulates synthesis of cGMP from GTP via retinal guanylate cyclase (RetGC) encoded by the retinal guanylate cyclase 2D (*GUCY2D*) gene^2,3^. GCAP-1 has four EF-hand domains, three (EF-2, EF-3, and EF-4) of which exhibit Ca^2+^/Mg^2+^ binding and act as Ca^2+^/Mg^2+^ sensor proteins^4–6^. Each EF-hand domain (EF-2, EF-3, and EF-4) consists of a helix-loop-helix secondary structure that is able to chelate Ca^2+^, whereas EF-1 domain serves as a target-binding domain. In the dark-adapted state, when cytosolic Ca^2+^ concentration is high, GCAP-1 binds Ca^2+^ and inhibits RetGC activity and cGMP synthesis. cGMP is hydrolyzed by light-activated phosphodiesterase in the light-adapted state, leading to closure of cation channels and low cytosolic Ca^2+^ concentration^7,8^ in which GCAP-1 releases Ca^2+^. Then, Mg^2+^ binds to the EF-2 and/or EF-3 in the Ca^2+^-free state of GCAP-1, and the Mg^2+^-bound GCAP-1 activates RetGC. Interestingly, mutated GCAP-1 (e.g. p.Y99C, p.D100E, p.L151F) associated with disease-causing *GUCA1A* variants show persistently stimulated RetGC activity, leading to the elevation of cytosolic Ca^2+^ and cGMP concentrations and resulting in initiation of photoreceptor cell death^9–13^.

Heterozygous *GUCA1A* variants have been reported as causes of autosomal dominant (AD) macular dystrophy (MD), cone dystrophy (COD), and cone-rod dystrophy (CORD)^9,14–18^. Previous studies have revealed that most patients with *GUCA1A* variants finally exhibit a CORD phenotype with progressive macular atrophy^14,15,19,20^. To date, all reported *GUCA1A* variants are missense or in-frame insertion/deletion types, according to the Human Gene Mutation Database (HGMD, http://www.hgmd.cf.ac.uk/). A large US cohort study of inherited retinal dystrophies (IRDs) has revealed the prevalence of *GUCA1A*-associated IRD is 0.7% (7/1000 families)^21^. However, to our knowledge, IRDs associated with *GUCA1A* variants have never been reported in the Japanese population^22^.

In this study, we identified rare *GUCA1A* variants from our whole exome sequencing (WES) database composed of IRD patients from a large Japanese cohort. The purpose of this study was to investigate the pathogenicity of rare *GUCA1A* variants and clinical and genetic features of *GUCA1A*-associated IRDs.

## Results

### Genetic analysis

**Figure 1:**
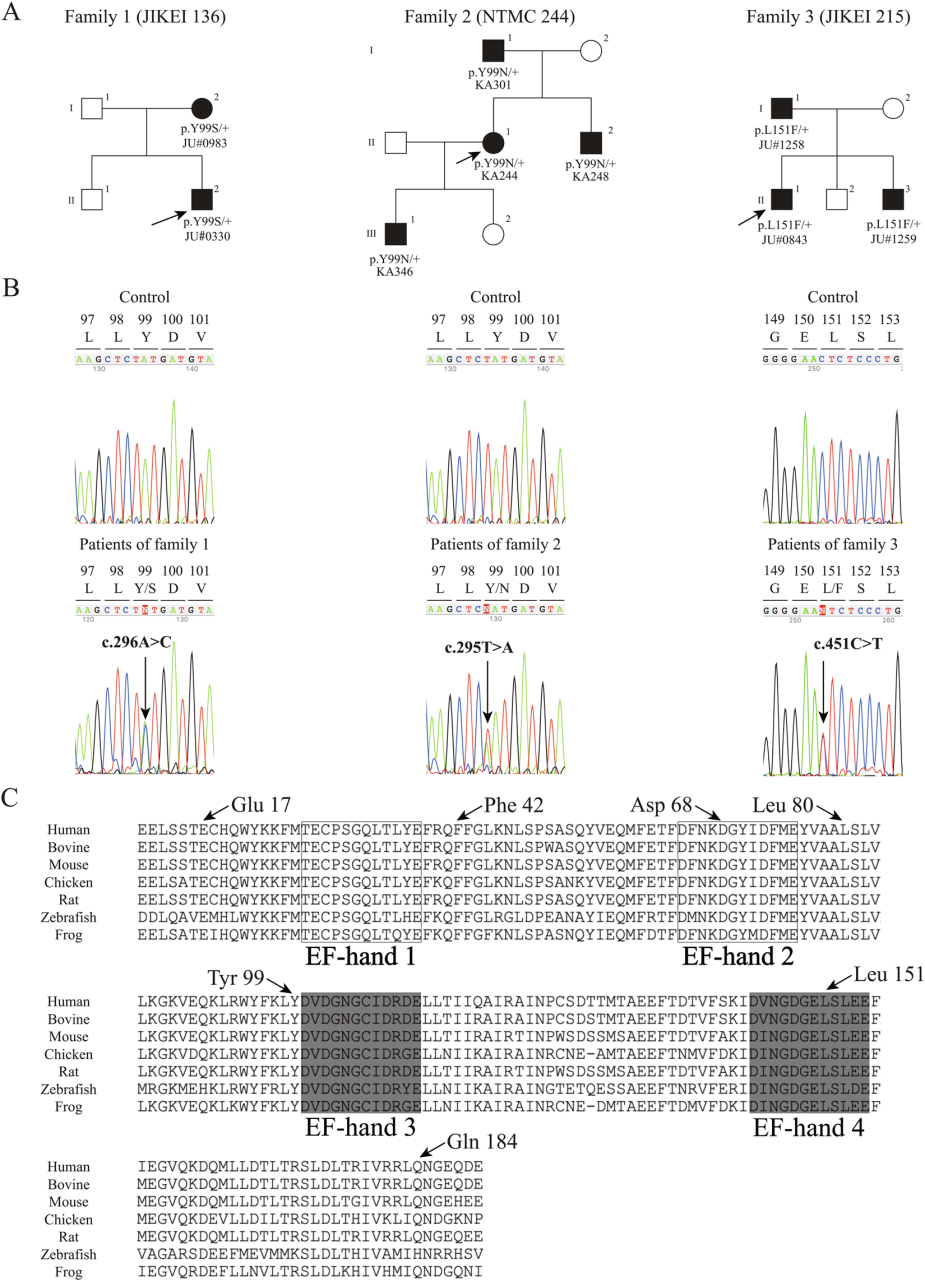
Pedigree charts of three Japanese families with *GUCA1A*-associated cone-rod dystrophy, nucleotide sequences of *GUCA1A* variants, and amino acid sequence alignment of GCAP-1 in different vertebrate species. (**A**) Solid squares (males) and circles (females) represent affected patients. Open squares (males) and circles (females) represent unaffected individuals. The proband of each family is indicated by an arrow. (**B**) Heterozygous variants [c.296 A > C (p.Y99S), c.295 T > A (p.Y99N), and c.451 C > T (p.L151F)] are shown in patients of families 1, 2, and 3, respectively. (**C**) The conserved 12-amino acid Ca^2+^ binding loop of EF-hand domains 3 and 4 are highlighted by gray shading. EF-hand domains 1 and 2 are surrounded by rectangles. Variants identified in this study are indicated by arrows.

We identified eight rare *GUCA1A* variants [c.C50_80del (p.E17VfsX22), c.T124A (p.F42I), c.C204G (p.D68E), c.C238A (p.L80I), c.T295A (p.Y99N), c.A296C (p.Y99S), c.C451T (p.L151F), and c.A551G (p.Q184R)] from 14 unrelated families after filtering (Supplementary Table S1). According to the American College of Medical Genetics and Genomics (ACMG) criteria, three variants (p.Y99N, p.Y99S, and p.L151F) from three families (NTMC 244, JIKEI 136, and JIKEI 215) were confirmed as “pathogenic” (Fig. 1A,B). Two (p.Y99N and p.Y99S) of the 3 variants were novel, while the remaining variant (p.L151F) was previously reported as the cause of AD-CORD^16,19^. The 3 variants co-segregated with the disease and were located on/around EF-hand domain 3 or 4, which is essential for cytosolic Ca^2+^/Mg^2+^ binding (Fig. 1C). *In silico* programs predicted severe damage to GCAP-1 (Supplementary Table S1), and each affected patient was diagnosed with COD/CORD. In our WES analysis, no other pathogenic variant, which is listed in the RetNet database, was found in the 3 families (NTMC 244, JIKEI 136, and JIKEI 215). In contrast, the other 5 variants (p.E17VfsX22, p.F42I, p.D68E, p.L80I, and p.Q184R) identified in 11 families were classified as “uncertain significance” according to the ACMG criteria (Supplementary Table S1). All 5 variants were located outside of EF-hand domains 3 and 4 (Fig. 1C) and did not segregate with the disease; therefore, these variants were considered to be “non-pathogenic”.

### Clinical study

In 3 families with the pathogenic *GUCA1A* variants (p.Y99S, p.Y99N, and p.L151F), a total of 9 affected patients were clinically investigated (Fig. 1A). The examined age ranged from 15 to 69 years old (mean age: 43.0 years, standard deviation: 18.3 years). Clinical findings of the 9 affected patients are summarized in Table 1.

**Table 1:** Clinical finidngs of patients with pathogenic variants.

Family ID, patient ID, Unique ID, age of onset, examined age, gender	Initial symptoms	BCVA (decimal)		Stage	Fundus photographs	Fundus autofluorescence imaging	Optical coherence tomography		Visual field testing (Goldmann perimetry)	Full-filed electroretinogram	*GUCA1A* variant
		RE	LE				EZ line	Thinning of outer retinal layers			
1, II-2, JU0330, 3, 40, M	Reduced visual acuity	0.2	0.2	Advanced stage	Extention of the retinal atrophy in direction to the optic disc and inferior arcade vessel	Loss of AF at retinal atrophy area with hyper-AF around the area	Disrupted	Present	Absolute central scotoma	Rod: decreased b-wave, Combined: normal a-wave and decreased b-wave, Cone and 30-Hz flickers: non-recordable	c.296 A > C (p.Y99S)
1, I-2, JU0983, 6, 68, F	Reduced visual acuity	0.01	0.02	Advanced stage	Extention of the retinal atrophy over the optic disc and inferior arcade vessel	Loss of AF at retinal atrophy area with hyper-AF around the area	Disrupted	Present	Loss of central visual field	Rod: non-recordable, Combined: non-recordable, Cone and 30-Hz flickers: non-recordable	c.296 A > C (p.Y99S)
2, II-1, KA244, 22, 35, F	Photophobia, reduced visual acuity	0.2	0.1	Middle stage	Macular atrophy	hypo-AF at retinal atrophy area with hyper-AF around the area	Disrupted/diffused	Absent	Absolute central scotoma	Rod: decreased b-wave, Combined: decreased a and b-waves, Cone and 30-Hz flickers: non-recordable	c.295 T > A (p.Y99N)
2, II-2, KA248, 19, 30, M	Photophobia, reduced visual acuity	0.15	0.1	Advanced stage	Severe macular atrophy	Loss of AF at retinal atrophy area with hyper-AF around the area	Disrupted	Present	Absolute central scotoma	Rod: decreased b-wave, Combined: decreased a and b-waves, Cone and 30-Hz flickers: non-recordable	c.295 T > A (p.Y99N)
2, I-1, KA301, 18, 65, M	Reduced visual acuity, central visual field loss	0.01	0.01	Advanced stage	Elliptical enlargement of retinal atrophy within the vascular arcades	Loss of AF at retinal atrophy area with hyper-AF around the area	Disrupted	Present	Not done	Not done	c.295 T > A (p.Y99N)
2, III-1, KA346, 4, 15, M	Photophobia and reduced visual acuity	0.7	0.7	Early stage	Discoloration limited at the fovea	Slight hyper-AF limited at the fovea	Preserved	Absent	Normal in RE and slightly decreased central sensitivity in LE in HFA	Rod: normal b-wave, Combined: normal a-wave and decreased b-wave, Cone and 30-Hz flickers: severely decreased	c.295 T > A (p.Y99N)
3, II-1, JU0843, 30, 34, M	Reduced visual acuity	0.5	0.8	Middle stage	Almost normal fundus appearance	Hyper-AF ring around the fovea	Disrupted/diffused	Absent	Relative central scotoma	Rod: normal b-wave, Combined: normal a-wave and decreased b-wave, Cone and 30-Hz flickers: severely decreased	c.451 C > T (p.L151F)
3, I-1, JU1258, NI, 69, M	Reduced visual acuity	0.09	0.06	Advanced stage	Elliptical enlargement of retinal atrophy in direction over optic disc to nasal retina	Loss of AF at retinal atrophy area with hyper-AF around the area	Disrupted	Present	Not done	Rod: decreased b-wave, Combined: decreased a and b-waves, Cone and 30-Hz flickers: non-recordable	c.451 C > T (p.L151F)
3, II-2, JU1259, NI, 31, M	Reduced visual acuity	0.2	0.4	Middle stage	Slight macular atrophy	Hyper-AF ring around the fovea	Disrupted/diffused	Absent	Absolute central scotoma in RE and relative central scotoma in LE	Rod: normal b-wave, Combined: normal a-wave and decreased b-wave, Cone and 30-Hz flickers: severely decreased	c.451 C > T (p.L151F)

AF = autofluorescence, BCVA = best corrected visual acuity, BE = both eyes, DA = dark adaptation, EZ = ellipsoid zone, F = female, HFA = Humphrey Field Analyzer, LA = light adaptation, LE = left eye, NI = no information, M = male, RE = right eye.

### Onset, initial symptoms, and visual acuity

The age of onset ranged from 3 to 30 years old (mean age: 14.6 years, standard deviation: 9.6 years). Initial symptoms were available from 9 patients and included reduced visual acuity (9/9, 100%), photophobia (3/9, 33.3%), and central visual field loss (1/9; 11.1%). The decimal best-corrected visual acuity of the nine patients ranged from 0.01 to 0.8 at the examined age (Table 1).

### Fundus photographs, FAF, and OCT findings

**Figure 2:**
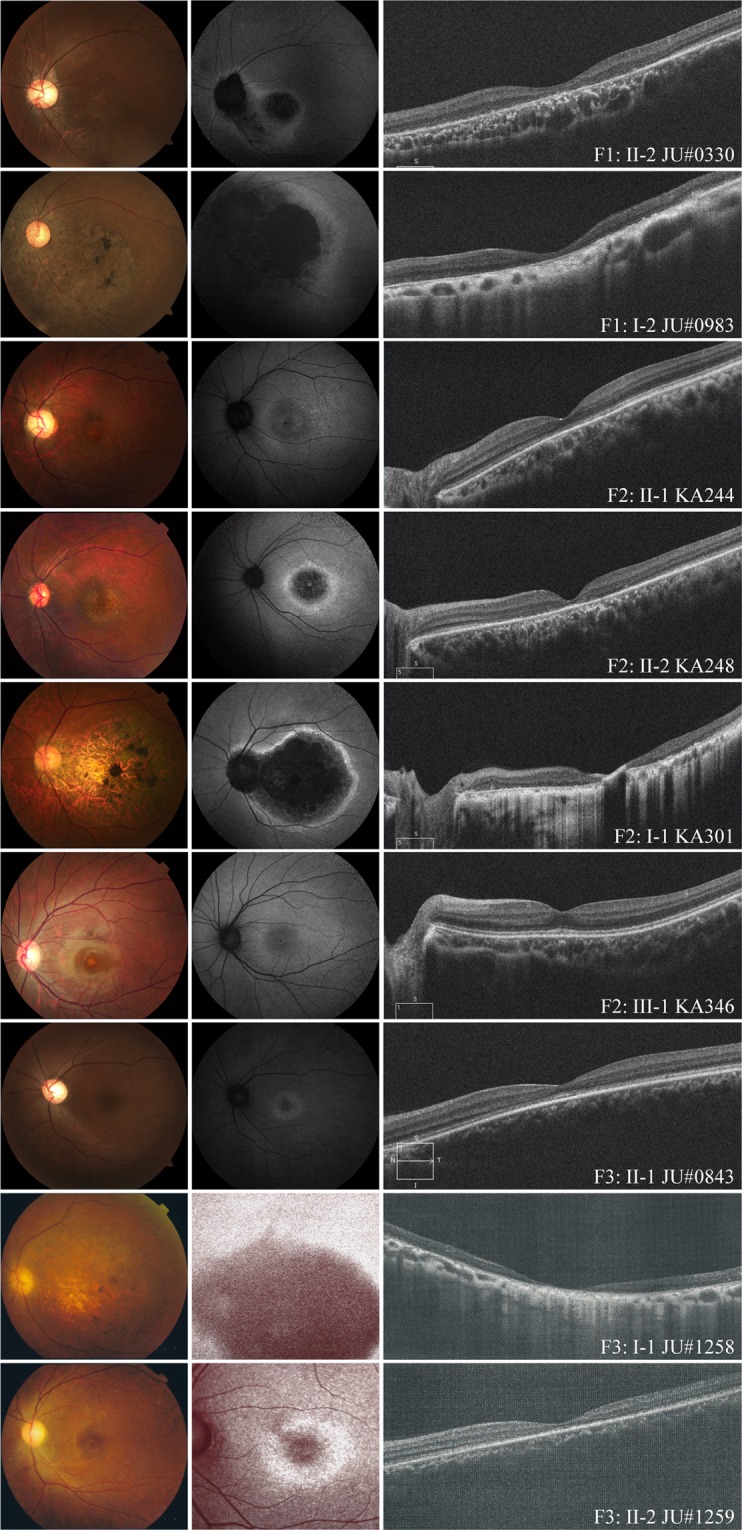
Fundus photographs, fundus autofluorescence imaging, and optical coherence tomography images. Each patient showed variable degrees of macular atrophy. Nine patients were classified into three stages (early stage: retinal abnormalities limited at the fovea, middle stage: retinal abnormalities within the macular area and advanced stage: retinal abnormalities beyond the macular area) based on the severity of macular atrophy. One patient (F2: III-1) had early stage abnormality, three patients (F3: II-1, F3: II-2, and F2: II-1) had middle stage abnormalities, and five patients (F1: II-2, F1: I-2, F2: II-2, F2: I-1, and F3: I-1) had advanced stage abnormalities.

Each examination showed variable degrees of retinal abnormalities (Fig. 2) and we classified the abnormalities into early, middle, and advanced stages according to the severity of macular atrophy. Patient F2: III-1 was classified into early stage and exhibited discoloration and slight hyper-autofluorescence limited at the fovea in fundus and FAF images and preserved hyper-reflectivity of the ellipsoid zone (EZ) with the foveal bulge in OCT images. Two patients (F3: II-1 and F3: II-2) were classified into middle stage and exhibited almost normal fundus appearance or slight macular atrophy in fundus photographs, hyper-autofluorescent ring around the fovea in FAF images, and diffusely decreased or disrupted EZ without the foveal bulge in OCT images. Patient F2: II-1 was also classified into middle stage and exhibited macular atrophy in fundus photographs, hypo-autofluorescent area corresponding to retinal atrophy with hyper-autofluorescence around the area in FAF images, and diffusely decreased or disrupted EZ without the foveal bulge in OCT images. Five patients (F1: II-2, F1: I-2, F2: II-2, F2: I-1, and F3: I-1) were classified into advanced stage and exhibited retinal atrophy at the macula or posterior pole in fundus photographs, loss of autofluorescent area corresponding to retinal atrophy with hyper-autofluorescence around the area in FAF images, and disrupted EZ and thinning of the outer retina corresponding to the retinal atrophic area with hypo-reflectivity of the EZ outside the area in OCT images.

The atrophic areas were apparently more enlarged in older patients (i.e., 15-year-old patient F2: III-1 at the early stage, mean age of 33.3 years in the middle stage, and mean age of 54.4 years in the advanced stage). In addition, the enlargement pattern of the atrophic area was different between family 1 and the other 2 families. Retinal atrophy in family 1 extended over the optic disc and inferior arcade whereas that in families 2 and 3 showed elliptical enlargement within the vascular arcade.

### Visual field findings

The results of visual field testing were obtained from 14 eyes of 7 patients. All 7 patients exhibited central visual field loss corresponding to retinal abnormalities. Patient F2: III-1 with early stage abnormality showed normal visual field in the right eye and slightly decreased central sensitivity in the left eye in HFA. The other six patients were examined using GP. Two patients with middle stage abnormalities showed central scotoma of V-4e isopter in the right eye (F3: II-2) and central scotoma of I-4e isopter in three eyes (both eyes of F3: II-1 and the left eye of F3: II-2). One patient with middle stage abnormality and two patients with advanced stage abnormalities showed central scotoma of V-4e isopter in all eyes (F2: II-1, F1: II-2, and F2: II-2), and one patient with advanced stage abnormality showed central visual field loss in both eyes (F1: I-2). The areas of central scotomas were consistent with the lesions of retinal atrophy.

Four (F1: II-2, F2: II-2, F3: II-1, and F3: II-2) of 5 patients, whose retinal atrophy was limited to the posterior pole, showed preserved peripheral visual fields. In contrast, patients F2: II-1 and F1: I-2 exhibited constriction of the peripheral visual field and an island limited to the inferior area.

### Full-filed electroretinographic findings

**Figure 3:**
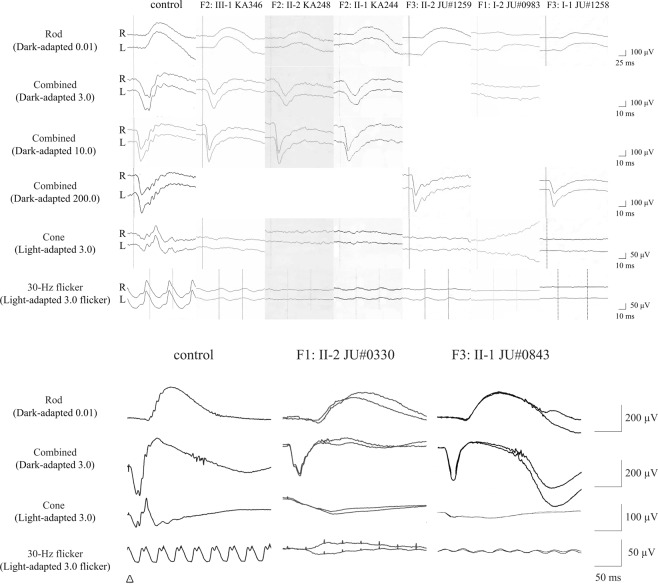
Full-field electroretinography findings. Full-field electroretinography (FF-ERG) shows severely decreased or non-recordable light-adapted cone and 30-Hz flicker responses in all eight examined patients. Dark-adapted rod and combined rod-cone/flash ERG findings show preserved responses in three patients (F2: III-1, F3: II-1, and F3: II-2), decreased responses in four patients (F1: II-2, F2: II-2, F2: II-1, and F3: I-1), and non-recordable responses in one patient (F1: I-2).

Full-field (FF)-electroretinography (ERG) recording was performed in 16 eyes of 8 patients. The results showed severely decreased or non-recordable cone responses in all patients (Fig. 3). Regarding rod responses, three patients (F2: III-1, F3: II-1, and F3: II-2) with early to middle stage abnormalities exhibited preserved rod responses, whereas 5 patients (F1: I-2, F1: II-2, F2: II-2, F2: II-1, and F3: I-1) with advanced stage abnormalities exhibited decreased and non-recordable rod responses, respectively (Fig. 3).

## Discussion

In this study, eight rare *GUCA1A* variants, three of which were pathogenic, were identified in our large Japanese cohort. Furthermore, we demonstrated clinical features of nine COD/CORD patients from three unrelated Japanese families with two novel variants (p.Y99S and p.Y99N) and one known variant (p.L151F).

To date, 19 *GUCA1A* missense and 3 in-frame deletion/insertion variants in heterozygous states have been reported as causes of AD-MD and AD-COD/CORD in HGMD Professional (2019.3)^14–18,20,23–33^. Most reported variants (18/22, 81.8%) were concentrated within or around EF-hand domains 3 and 4, which are essential for cytosolic Ca^2+^/Mg^2+^ binding^4–6^. Amino acid residues Y99 and L151 are also located on EF-hand 3 helix E and EF-hand 4 loop, respectively (Fig. 1C). Regarding the two novel variants (p.Y99S and p.Y99N), a different variant (p.Y99C) at the same position has been reported as “pathogenic” and leads to COD/CORD^14,34^. The variant p.L151F has also been reported as a cause of CORD^16,19^. In contrast, the other five variants (p.E17VfsX22, p.F42I, p.D68E, p.L80I, and p.Q184R) identified in the present study, which resulted in “uncertain significance” in the ACMG criteria, did not co-segregate with the disease. Previously, only five missense variants (p.L50I, p.L84F, p.G86R, p.E89K, and p.L176F) located outside EF-hand domains 3 and 4 have been reported as “pathogenic”,^14,15,18,23,33^ and one variant (p.L50I) has been concluded as “non-pathogenic” by subsequent studies^25,35^. Experimental studies have clarified that most *GUCA1A* variants within or around EF-hand domains 3 and 4 lead to the constitutive activation of RetGC by mechanisms of either a dominant-negative effect or gain of function, not haploinsufficiency^9,19,23,27,30,31,36^. While, recent studies have also demonstrated that the mutated GCAP-1 (p.L84F and p.L176F), located outside EF-hand domains 3 and 4, showed a significantly high affinity for Mg^2+^ by altered tertiary structure or conformational changes, resulting in stabilizing the RetGC-activating state^36,37^. Certainly, all reported *GUCA1A* variants are heterozygous missense or in-frame insertion/deletion variants, but not truncated variants, compatible with the mechanisms of dominant-negative effect/gain of function. Regarding the reported p.Y99C variant^34^, the mutated GCAP-1 disrupts the N-terminal helix of the helix-loop-helix conformation of the EF-hand domain 3, severely affecting Ca^2+^ binding at the site^9,10^. Further, the p.Y99C mutant activates RetGC at low Ca^2+^ concentration, similar to wild-type GCAP-1, but remains active even at high Ca^2+^ concentration, when wild-type GCAP-1 normally inhibits the target^9,10,13^. A similar pathomechanism whereby RetGC activity cannot be suppressed even at high cytosol Ca^2+^ concentration may be considered to be CORD caused by our novel variants (p.Y99N and p.Y99S). While it is demonstrated that the p.L151F mutant showed decreased Ca^2+^ affinity and significantly lower thermal stability compared to the wild-type protein^38^. Among the five non-pathogenic *GUCA1A* variants identified in this study, p.E17VfsX22, a truncated variant, might be considered to be a haploinsufficiency mechanism, whereas the other four missense variants were located outside EF-hand domains 3 or 4. However, we did not perform any biochemical or biophysical experiment using the four missense mutatns (p.F42I, p.D68E, p.L80I, and p.Q184R). Further, complex pathogenic mechanisms have been reported about mutated GCAP-1 outside EF-hand domains 3 and 4^18,36,37^. Although functional influence by the mutated GCAP-1 was not determined experimentally, all five *GUCA1A* variants (p.E17VfsX22, p.F42I, p.D68E, p.L80I, and p.Q184R) did not co-segregate with the disease; some family members with the variants were unaffected, concluding that the 5 variants were non-pathogenic.

All reported *GUCA1A*-associated phenotypes are classified into AD-MD or AD-COD/CORD with progressive macular atrophy^14,15,19,23,25,32^. In our study, the retinal atrophic areas were apparently more enlarged in older patients. In regard to cone ERG, although previous studies have shown that a variable degree of responses is seen ranging from preserved cone responses in AD-MD to severely decreased cone responses in AD-COD/CORD^14,19,23,25,32^, all 8 patients examined exhibited severely decreased or non-recordable cone responses (Fig. 3). In contrast, the findings of rod ERG showed a variable degree of responses ranging from nearly normal to non-recordable (Fig. 3), consistent with data from previous studies^14,15,19,20,23–25,30,31^. It is unclear why cone photoreceptors are initially and predominantly affected in *GUCA1A*-associated COD/CORD. In rod photoreceptors of mice, both GCAP-1 and GCAP-2 (an isoform of GCAP-1) are expressed, whereas GCAP-1 is predominantly expressed in cone photoreceptors^39^. Thus, GCAP-2 could compensate for dysfunction of GCAP-1 caused by *GUCA1A* variants in human rod photoreceptors as pointed out previously^40^. Another possibility is that GCAP-2 reduces an abnormal RetGC activity through competition with mutated GCAP-1 in which wild-type versus mutated GCPA-1 might function at different ratios between cone and rod photoreceptors. Taken together, retinal function gradually deteriorates and leads to COD/CORD in patients with heterozygous pathogenic *GUCA1A* variants. Clinical features of our 9 patients were consistent with previous studies as CORD with progressive macular atrophy; however, the centrifugal extension pattern of retinal atrophy was different between the families (Fig. 2). Unlike elliptical enlargement of retinal atrophy in families 2 and 3 and previous studies^11–13^, retinal atrophy in family 1 extended over the optic disc and inferior arcade. This unique pattern of retinal atrophy might be a characteristic finding of the p.Y99S variant or be influenced by secondary (genetic or environmental) factors. In addition, the older patient (F1: I-2) with p.Y99S showed non-recordable responses not only in cone ERG but also in rod and combined ERG, and peripheral visual field loss except for the inferior area. To our knowledge, this is the first report of such a case. The clinical course of *GUCA1A*-associated dystrophies typically exhibits progressive cone dysfunction with macular atrophy, accompanied by later rod dysfunction. Although it is not clarified whether *GUCA1A*-associated dystrophies ultimately lead to entire loss of rod function in addition to loss of cone function, our finding of patient F1: I-2 suggests that these dystrophies might lead to severe loss of both rod and cone function as the result of continuous progression.

In this study, our cohort of IRDs revealed the prevalence (0.25%, 3/1192 families) of *GUCA1A*-associated IRDs was lower than that (0.7%, 7/1000 families) of previous large US cohort study^21^. Our study suggests that *GUCA1A*-associated IRDs in Japanese population may be rarer compared with US population. In fact, any IRDs associated with *GUCA1A* variants have never been reported in the Japanese population. Further investigations will need to be undertaken in order to clarify the prevalence of *GUCA1A*-associated IRDs in each population.

In conclusion, we identified eight rare *GUCA1A* variants including three pathogenic variants from a large Japanese cohort. Our results indicated that the three pathogenic variants underlie AD-COD/CORD with progressive retinal atrophy, and the prevalence of *GUCA1A*-associated IRDs may be low among Japanese patients with IRDs.

## Patients and Methods

### Ethics statement

Institutional Review Boards of the seven participating institutions approved the study protocol [The Jikei University School of Medicine (approval number 24–232 6997); National Hospital Organization Tokyo Medical Center (approval number: R18-029); Nagoya University Graduate School of Medicine (approval number: 2010–1067); Mie University Graduate School of Medicine (approval number: 2429); Teikyo University School of Medicine (approval number: 10-007-4); Iwate Medical University School of Medicine (approval number: HGH23-1); and Kindai University Faculty of Medicine (approval number: 22–132)]. The protocol adhered to the tenets of the Declaration of Helsinki, and informed consent was obtained from each participant before participating in this study.

### Molecular genetic study

Patients with IRDs were studied from the genotype-phenotype database of the Japan Eye Genetics Consortium (http://www.jegc.org/)^41,42^. We examined our in-house WES database of 1385 IRD patients and 682 of their family members from 1192 Japanese families. In fact, WES with targeted analysis of the IRD genes listed in the RetNet database (https://sph.uth.edu/retnet/home.htm) was performed.The details of the WES methodology are described elsewhere^41,43^. We selected patients with IRDs who had rare *GUCA1A* variants in at least in one allele with a frequency of less than 1% in the Human Genetic Variation Database (http://www.hgvd.genome.med.kyoto-u.ac.jp/index.html) and a total frequency of less than 1.0% of the genome Aggregation Database (http://gnomad.broadinstitute.org). Subsequently, the pathogenicity of identified *GUCA1A* variants was evaluated according to the standards and guidelines of the ACMG^44^. Lastly, we evaluated the damage to GCAP-1 by using three *in silico* programs [PolyPhen2 (http://genetics.bwh.harvard.edu/pph2/), SIFT (http://sift.jcvi.org/www/SIFT_seq_submit2.html), and MutationTaster (http://www.mutationtaster.org/)]. Confirmation and segregation of each *GUCA1A* variant were performed by Sanger sequencing. Identified *GUCA1A* variants were compared with the NCBI reference sequence (NM_ 000409.4).

### Clinical study

We retrospectively reviewed the detailed medical records of patients with *GUCA1A* variants, which were determined as pathogenic in genetic analysis. Each patient underwent a comprehensive ophthalmic examination, including decimal best-corrected visual acuity, funduscopy, fundus autofluorescence imaging (FAF; Spectralis HRA; Heidelberg Engineering, Heidelberg, Germany), optical coherence tomography (OCT; Carl Zeiss Meditec AG, Dublin, CA, USA), and visual field testing using Goldmann perimetry (GP; Haag-Streit, Bern, Switzerland) and/or Humphrey field analyzer (HFA; Carl Zeiss Meditec AG). FF-ERG using a light-emitting diode built-in electrode (LE-4000, Tomey, Nagoya, Japan) was recorded in accordance with the protocols of the International Society for Clinical Electrophysiology of Vision (ISCEV)^45^ with the exception of using a stronger flash under dark-adapted (DA) conditions to record a DA 200 (200 cd·s·m^−2^) ERG. Ganzfeld FF-ERG using Neuropack 2 (Nihon Kohden, Tokyo, Japan) with corneal contact lens electrodes was recorded according to the ISCEV protocols except for DA 10.0 (10.0 cd·s·m^−2^) ERG. The detailed procedure and conditions of FF-ERG were previously reported^41,46–48^.

## Supplementary information

Supplementary Table S1
