# Supplementary material for: Characterization of GUCA1A-associated dominant cone/cone-rod dystrophy: low prevalence among Japanese patients with inherited retinal dystrophies
Source: Sci Rep. 2019 Nov 14;9:16851. doi: 10.1038/s41598-019-52660-1 (PMC6856191; doi:10.1038/s41598-019-52660-1)
Supplement: Supplementary file 1 — Supplementary Table S1 [file 41598_2019_52660_MOESM1_ESM.docx]

Supplementary Table S1 Evaluation of identified rare *GUCA1A* variants in this study

| Exon | Nucleotide sequence | Amino acid sequence | dbSNP ID | Frequency in database (%) | | | | | | | *In silico* analysis | | | Family | Segregation results | The number of normal cases with *GUCA1A* variant | Domains | ACMG classification | |
| --- | --- | --- | --- | --- | --- | --- | --- | --- | --- | --- | --- | --- | --- | --- | --- | --- | --- | --- | --- |
|  |  |  |  | HGVD | ToMMo | GnomAD, East Asia | GnomAD, South Asia | GnomAD, African | GnomAD, European  (non-Finnish) | GnomAD, Total | Polyphen-2 (HumDiv) | SIFT | Mutation taster |  |  |  |  | Identified criteria | Classification |
| 3 | c.C50_80del | p.E17VfsX22 | Not repoted | Not reported | Not reported | Not reported | Not reported | Not reported | Not reported | Not reported |  |  |  | IWATE003  JIKEI010 | Not matched | 3 | EF-1 domain | PM2, BS2, BS4 | Uncertain significance |
| 3 | c.124T>A | p.F42I | rs202098005 | Not reported | Not reported | 0.000 | 0.000 | 0.000 | 0.000 | 0.000 | Probably damaging | Tolerated | Disease causing | NTMC223  NAGOYA114 | NTMC223; not matched  NAGOYA114; not done | 2 | EF-1 domain | PM2, BS2, BS4 | Uncertain significance |
| 3 | c.204C>G | p.D68E | rs776251040 | 0.0511 | Not reported | 0.000 | 0.006 | 0.000 | 0.000 | 0.000 | Probably damaging | Damaging | Disease causing | NTMC262 | Not matched | 1 | EF-2 loop | PM2, BS2, BS4 | Uncertain significance |
| 4 | c.238C>A | p.L80I | Not repoted | Not reported | Not reported | Not reported | Not reported | Not reported | Not reported | Not reported | Probably damaging | Damaging | Disease causing | TEIKYO009 | Not matched | 2 | EF-2 domain | PM2, BS2, BS4 | Uncertain significance |
| 4 | c.295T>A | p.Y99N | Not repoted | Not reported | Not reported | Not reported | Not reported | Not reported | Not reported | Not reported | Probably damaging | Damaging | Disease causing | NTMC244 | Matched | 0 | EF-3 helix E | PS3, PM1, PM2, PM5, PP1 | Pathogenic |
| 4 | c.296A>C | p.Y99S | Not repoted | Not reported | Not reported | Not reported | Not reported | Not reported | Not reported | Not reported | Probably damaging | Damaging | Disease causing | JIKEI136 | Matched | 0 | EF-3 helix E | PS3, PM1, PM2, PM5, PP1 | Pathogenic |
| 6 | c.451C>T | p.L151F | rs121434631 | Not reported | Not reported | 0.000 | 0.000 | 0.006 | 0.000 | 0.000 | Probably damaging | Damaging | Disease causing | JIKEI215 | Matched | 0 | EF-4 loop | PS1, PS3, PM1, PM2, PP1 | Pathogenic |
| 6 | c.551A>G | p.Q184R | rs149998844 | 0.0544 | Not reported | 0.131 | 0.000 | 0.000 | 0.000 | 0.009 | Benign | Damaging | Disease causing | JIKEI118, NTMC234, NTMC290, KINKI051, MIE066 | Two families; not matched, Remained 3 families; not done | 2 | Outside EF domains | PM2, BS2, BS4 | Uncertain significance |

"ACMG, the standards and guidelines of the American College of Medical Genetics and Genomics; db SNP, Single Nucleotide Polymorphism Database (https://www.ncbi.nlm.nih.gov/snp/); Gnom AD, Genome Aggregation Database (http:// gnomad.broadinstitute.org)

HGVD, Human Genetic Variation Database (http://www.hgvd.genome.med.kyoto-u.ac.jp/index.html); Mutation taster (http://www.mutationtaster.org/); Polyphen-2 (http://genetics.bwh.harvard.edu/pph2/); SIFT (http://sift.jcvi.org/www/SIFT_seq_submit2.html)

ToMMo, The Tohoku Medical Megabank Organization of Tohoku University (https://ijgvd.megabank.tohoku.ac.jp)"
